# Safflower Extract Inhibits ADP-Induced Human Platelet Aggregation

**DOI:** 10.3390/plants10061192

**Published:** 2021-06-11

**Authors:** Ping-Hsun Lu, Chan-Yen Kuo, Chuan-Chi Chan, Lu-Kai Wang, Mao-Liang Chen, I-Shiang Tzeng, Fu-Ming Tsai

**Affiliations:** 1 Department of Chinese Medicine, Taipei Tzu Chi Hospital, The Buddhist Tzu Chi Medical Foundation, New Taipei City 231, Taiwan; pinghsunlu@gmail.com; 2 School of Post-Baccalaureate Chinese Medicine, Tzu Chi University, Hualien 970, Taiwan; 3 Department of Research, Taipei Tzu Chi Hospital, The Buddhist Tzu Chi Medical Foundation, New Taipei City 231, Taiwan; cykuo863135@gmail.com (C.-Y.K.); cater0656@hotmail.com (M.-L.C.); istzeng@gmail.com (I.-S.T.); 4 Department of Laboratory Medicine, Taipei Tzu Chi Hospital, The Buddhist Tzu Chi Medical Foundation, New Taipei City 231, Taiwan; kiki1205@tzuchi.com.tw; 5 Radiation Biology Core Laboratory, Institute for Radiological Research, Chang Gung University/Chang Gung Memorial Hospital, Linkou, Taoyuan 333, Taiwan; lukai2724@cgmh.org.tw

**Keywords:** safflower, hydroxysafflor yellow A, safflower yellow A, flavonoid, platelet aggregation

## Abstract

Safflower extract is commonly used as a traditional Chinese medicine to promote blood circulation and remove blood stasis. The antioxidant and anticancer properties of safflower extracts have been extensively studied, but their antiaggregative effects have been less analyzed. We found that safflower extract inhibited human platelet aggregation induced by ADP. In addition, we further analyzed several safflower extract compounds, such as hydroxysafflor yellow A, safflower yellow A, and luteolin, which have the same antiaggregative effect. In addition to analyzing the active components of the safflower extract, we also analyzed their roles in the ADP signaling pathways. Safflower extract can affect the activation of downstream conductors of ADP receptors (such as the production of calcium ions and cAMP), thereby affecting the expression of activated glycoproteins on the platelet membrane and inhibiting platelet aggregation. According to the results of this study, the effect of safflower extract on promoting blood circulation and removing blood stasis may be related to its direct inhibition of platelet activation.

## 1. Introduction

Safflower, also known as thorn flower, is a type of chrysanthemum. The leaves are linear, thin, and long, with a special aroma and a bitter taste. Safflower can be grown in large areas and is less likely to die [1], so its survival rate is much higher than that of other plants. The safflower extract is obtained from the dried flowers of *Carthamustinctorius* L., a plant of the Asteraceae family, by removing impurities, drying in the shade, or drying under low heat. Safflower is often used as a traditional Chinese medicine to promote hormone balance, remove blemishes, regenerate new cells, circulate blood, remove bruises, and relieve pain [2,3,4].

The biological activity of the extract of safflower is related to the various compounds in the extract. Previous studies have indicated that the extract of safflower could be divided into lipophilic compounds and hydrophilic compounds due to different extraction methods [3]. The lipophilic compounds of safflower include fatty acids, tocopherols such as vitamin E, carotenoids, and phytosterols. Hydrophilic compounds include flavonoids such as safflower yellow A or hydroxysafflor yellow A, saponins, and other safflower nutrients such as sugars and amino acids. According to related studies of compounds in safflower, safflower has anti-inflammatory [5,6,7], anticancer, and antioxidation effects [8,9,10,11] and can also be used as a potential treatment for osteoporosis and brain and liver diseases [12,13,14,15,16].

Platelets are formed from the cytoplasm of mature megakaryocytes in the bone marrow. Each megakaryocyte can produce 2000–7000 platelets. Platelets have a half-life of approximately 7 to 9 days and are cleared mainly by macrophages in the spleen. Under normal circumstances, platelets are biconvex, disc-shaped, can extend out a foot process, and appear irregular after stimulation [17,18,19]. The main physiological function of platelets is hemostasis. In general, hemostasis can be divided into three processes. First, the platelets adhere to the outer side of the truncated endothelium, a process known as “adhesion.” Second, platelets change shape, turn on receptors, and secrete a chemical messenger is the process of “activation”. Finally, platelets bridge each other through receptors, a process known as “aggregation”. The formation of platelet emboli is related to the activation of coagulation factors and the production, deposition, and linkage of fibrin [20,21].

The processes of platelet adhesion, activation, and aggregation appear almost simultaneously after platelet activation. The adhesion of platelets refers to the adhesion of platelets and nonplatelet surfaces. The main substances involved in this process are the glycoproteins on the platelet membrane, the Von Willebrand factor in the plasma, and the collagen component in the subcutaneous tissue [22,23]. Aggregation refers to the adhesion of platelets to each other. The main substances that cause platelet aggregation are thromboxane A2 (TXA2), collagen, thrombin, and ADP [24,25]. Among them, ADP plays the most important role in inducing platelet aggregation physiologically, especially endogenous ADP released by platelets [26]. In vitro, a small amount of ADP (less than 0.9 μM) can induce platelet aggregation rapidly, but they depolymerize quickly. When a moderate amount of ADP (approximately 1 μM) was added, it could aggregate and deaggregate platelets, which could then quickly enter the second stage of the irreversible aggregation reaction. The irreversible aggregation reaction in the second stage is mainly caused by the release of endogenous ADP from platelets. If a large amount of ADP (approximately 10 μM) is added, the platelets go directly to the second stage of irreversible aggregation [27,28]. Generally, drugs that induce platelet aggregation will decrease the expression of cyclic AMP (cAMP) in platelets, which may be related to the increase in calcium ion concentration and release of endogenous ADP when the expression of cAMP in platelets decreases.

Many of the above studies have pointed to safflower’s role in anti-inflammation and inhibiting cancer cell growth. However, safflower is often used in traditional Chinese medicine to promote blood circulation and remove blood stasis. The mechanism of safflower in platelet aggregation remains unclear. In this study, we confirmed that safflower has antiplatelet aggregation activity by using ADP-induced human platelet aggregation experiments. In addition, we further analyzed the active compounds of the safflower extract and the effect of the safflower extract on the signal transduction of ADP-induced platelet aggregation.

## 2. Results

### 2.1. Safflower Extract Inhibited ADP-Induced Human Platelet Aggregation

**Figure 1:**
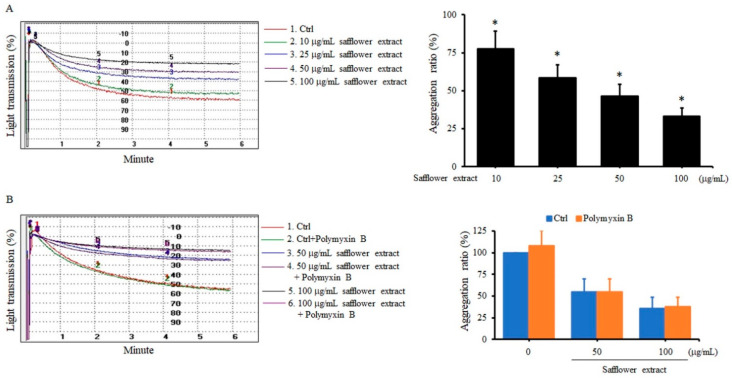
Safflower extract inhibited platelet aggregation. PRP was treated with different concentrations of safflower extract (**A**) or treated with 50–100 μg/mL safflower extract and polymyxin B (**B**). ADP was added and analyzed by an aggregometer. * *p* < 0.05 compared with the control group.

The results in Figure 1A show that as the dose of safflower extract increased, safflower inhibited ADP-induced human platelet aggregation in a dose-dependent manner. The inhibitory effect of safflower on platelet aggregation was 22.3–66.6%, and safflower at a dose of 100 μg/mL had the highest inhibitory effect. Many traditional Chinese medicine extracts interfere with bacterial endotoxin, so it is necessary to clarify whether endotoxin affects the platelet aggregation reaction in response to safflower extract. Polymyxin B, an inhibitor of Toll-like receptor 4, was used to observe whether endotoxin would affect the inhibition of platelet aggregation activity of the safflower extract. When platelets were treated with 50 μg/mL or 100 μg/mL safflower extract, ADP-induced platelet aggregation was significantly inhibited. When polymyxin B was added to platelets at the same time, the inhibition of ADP-induced platelet aggregation by safflower extract did not significantly change, indicating that safflower extract itself had the effect of platelet inhibition (Figure 1B). A dose of 50 μg/mL safflower extract was selected for the subsequent experiments.

### 2.2. All Compounds Found in Safflower Inhibited ADP-Induced Human Platelet Aggregation

**Figure 2:**
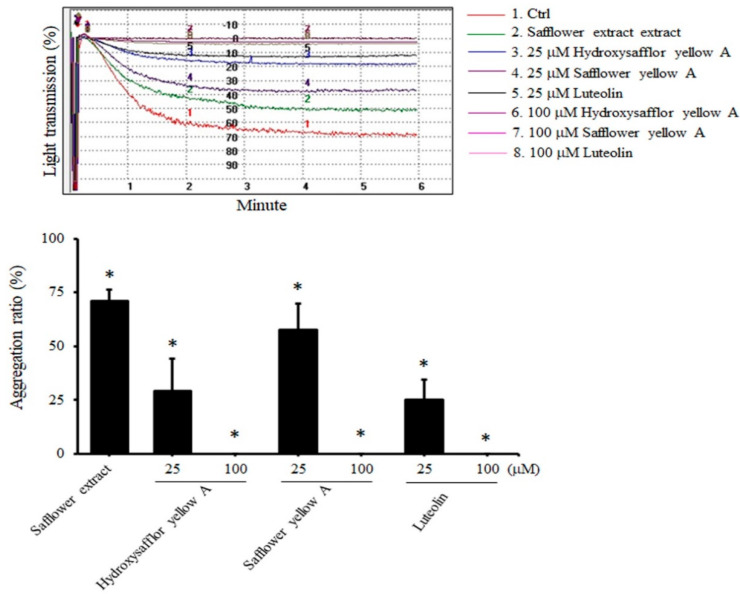
The compounds found in the safflower extract inhibited platelet aggregation. PRP was treated with 50 μg/mL safflower extract or 25–100 μg/mL compounds found in the safflower extract, and ADP was added and analyzed by an aggregometer. * *p* < 0.05 compared with the control group.

Since safflower extract inhibits platelet aggregation, we next wanted to explore which safflower extract compounds play an important role, and we selected three compounds available for further analysis. When human platelets were treated with 25 μM hydroxysafflor yellow A, safflower yellow A, or luteolin, ADP-induced platelet aggregation was significantly inhibited by 70.9%, 42.3%, and 74.9%, respectively. When platelets were treated with each high concentration (100 μM) of compound found in the safflower extract, almost no platelet aggregation reaction was found (Figure 2). These above results showed that these compounds found in safflower extract all have a strong antiplatelet aggregation effect. A dose of 25 μM of each compound found in the safflower extract was chosen for follow-up research.

### 2.3. Safflower Extract and Its Compounds Inhibit Platelet Aggregation through P2Y1 and P2Y12 Receptors

**Figure 3:**
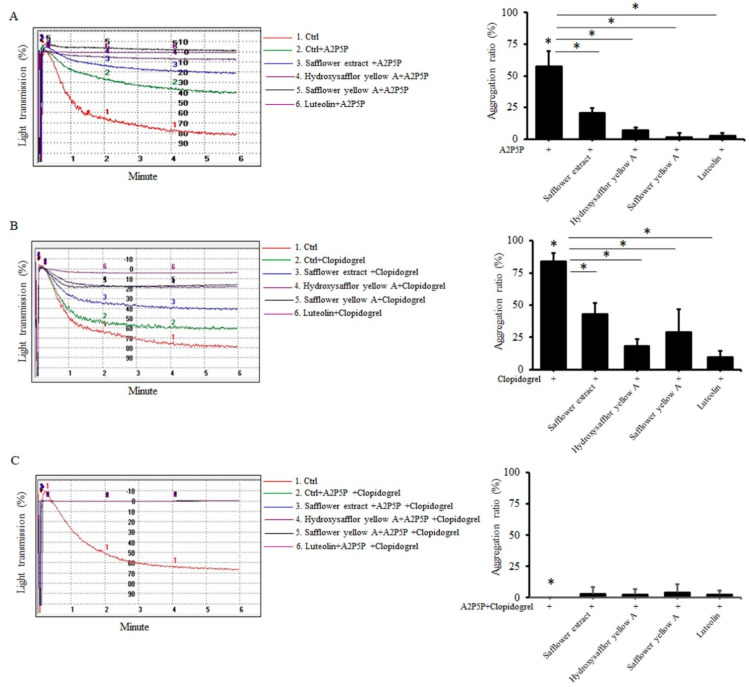
The effect of the ADP receptor antagonist on the inhibition of platelet aggregation by safflower extract. PRP was treated with 50 μg/mL safflower extract or 25 μg/mL of compounds found in safflower extract, 10 μM ADP and 1 mM A2P5P (**A**), 1 mM clopidogrel (**B**), or A2P5P+clopidogrel (**C**) added and analyzed by an aggregation analyzer. * *p* < 0.05 between two groups.

ADP induces platelet aggregation mainly through P2Y1 and P2Y12 receptors on the platelet membrane [26]. Next, we wanted to understand which receptor on platelets the safflower extract mainly targeted to inhibit platelet aggregation. When platelets were treated with the P2Y1 antagonist A2P5P, A2P5P significantly inhibited platelet aggregation by 42.3%, and the safflower extract and its compounds combined with A2P5P further inhibited ADP-induced platelet aggregation by 79.5–98.3% (Figure 3A), which means that the safflower extract and its compounds inhibited platelet aggregation through the P2Y12 pathway. Similarly, when platelets were treated with the P2Y12 antagonist clopidogrel, clopidogrel slightly inhibited platelet aggregation by 16%, and safflower extract and its compounds combined with clopidogrel further inhibited ADP-induced platelet aggregation by 57.1–90.3% (Figure 3B), which indicated that safflower extract and its compounds can also inhibit platelet aggregation through the P2Y1 pathway. When platelets were treated with A2P5P and clopidogrel at the same time, no aggregation reaction occurred (Figure 3C), confirming the importance of P2Y1 and P2Y12 receptors in the induction of platelets by ADP.

### 2.4. Safflower Extract and Its Compounds Inhibit the Activation of Calcium Ion Influx and the Production of cAMP Regulated by ADP

**Figure 4:**
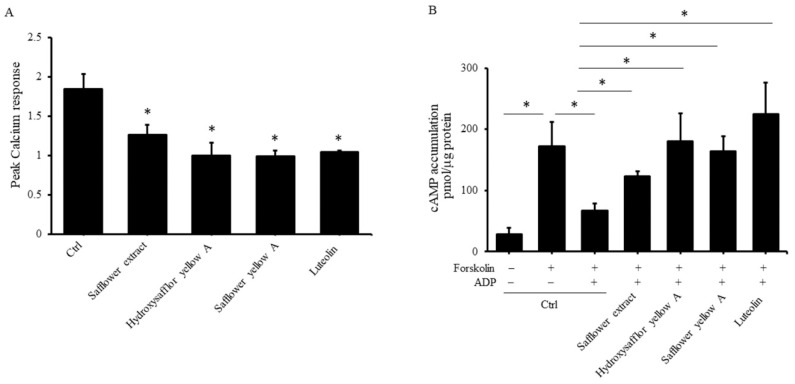
Effects of safflower extract on the production of calcium ions and cAMP in platelets. (**A**) PRP was treated with safflower extract or compounds found in safflower extract for 1 h and then reacted with Fura-2AM at 37 °C. Isolated platelets were collected, and calcium responses induced by ADP were measured. (**B**) PRP was treated with safflower extract or compounds found in safflower extract for 1 h. The purified platelets were collected and reacted with 10 μM forskolin and 10 μM ADP at 37 °C. Cell lysates were prepared, and the cAMP levels were measured by enzyme immunoassay. * *p* < 0.05 between two groups.

The results of experiments using the abovementioned antagonists found that safflower extract and its compounds inhibit the activation of P2Y1 and P2Y12 receptors on the platelet membrane. Next, we wanted to explore whether safflower extract and its compounds truly affect the downstream activation pathways involved in P2Y1 and P2Y12 receptors. We directly analyzed the safflower extract, and its compounds affected the activation of calcium influx induced by the P2Y1 receptor and cAMP production regulated by the P2Y12 receptor. ADP induced an increase in intracellular calcium ions in platelets by 1.84-fold, while safflower extract and its compounds inhibited the increase in calcium ion concentration induced by ADP by 31.3–46.4% (Figure 4A). When platelets were treated with forskolin, the cAMP concentration in platelets was significantly increased by 6.12-fold, and when platelets were added to ADP, the cAMP concentration in platelets was significantly decreased by 61.4%. When the platelets were exposed to safflower extract and its compounds at the same time, the decrease in cAMP production caused by ADP increased significantly by 84.5–237.9% (Figure 4B). The above results show that the safflower extract and its units can inhibit the participation of P2Y1 and P2Y12 in internal signal transduction in platelets.

### 2.5. Safflower Extract and Its Compounds Inhibit the Production of Thromboxane A2 (TXA2) and Arachidonic Acid (AA)

**Figure 5:**
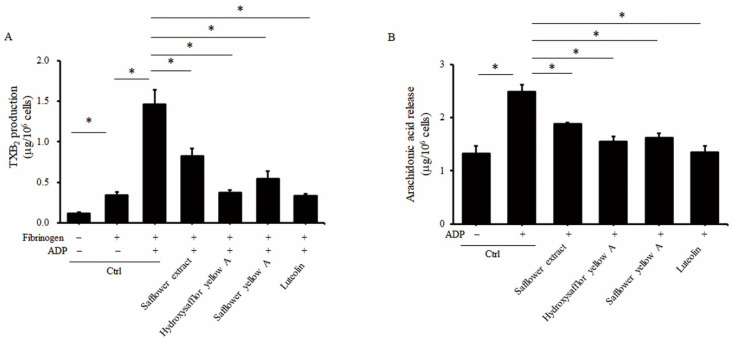
Effects of safflower extract on the production of TXB_2_ and AA in human platelets. PRP was treated with safflower extract or compounds found in safflower extract for 1 h. Isolated platelets were collected and then stimulated with 3 μM fibrinogen and 10 μM ADP at 37 °C. The content of TXB2 (**A**) or AA (**B**) was determined by enzyme immunoassay. * *p* < 0.05 between two groups.

After participating in calcium ion activation and regulation of cAMP production on the platelet membrane through the ADP receptor, the phospholipid on the membrane is then degraded, and AA is released, which then generates TXA2 and induces the formation of glycoprotein on the membrane to participate in the aggregation of platelets [29]. Therefore, in addition to calcium ion activation and cAMP production, we also wanted to explore whether safflower extract and its compounds could also affect the production of TXA2 and AA in platelets. Because of the poor stability of TXA2, TXB2 production was used to reflect the expression level of TXA2 in platelets. As shown in Figure 5A, fibrinogen increased the expression of TXB2 in platelets, and the addition of ADP had an additive effect. Compared with untreated platelets, TXB2 was increased 13.4-fold when platelets were treated with fibrinogen and ADP. However, when safflower extract or its compounds were treated with platelets at the same time, the increased yields of TXB2 induced by fibrinogen and ADP were significantly inhibited by 39.4–73%. In terms of AA production, fibrinogen and ADP can induce platelets to produce AA by 97.2%. Similarly, when platelets were simultaneously treated with safflower extract or its compounds, the production of AA was inhibited by 21.1–47.3% (Figure 5B).

### 2.6. Safflower Extract and Its Compounds Inhibit the Formation of PAC-1 in Platelets

**Figure 6:**
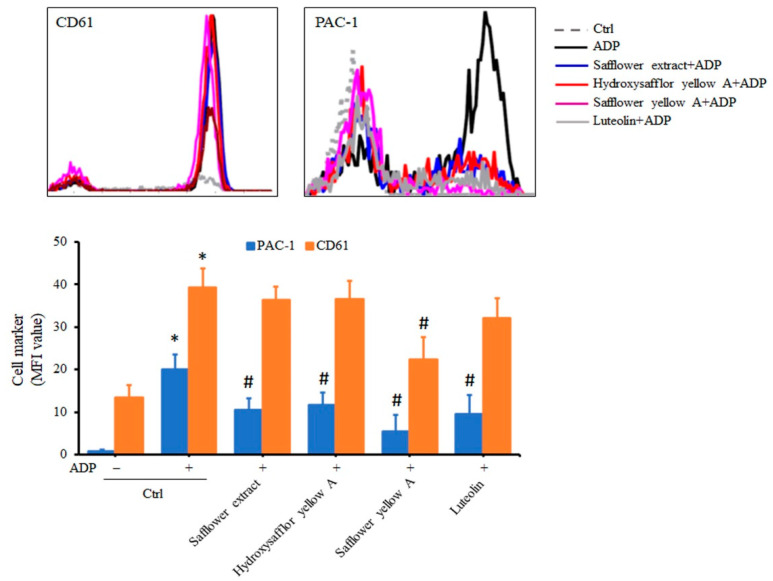
Effects of safflower extract on the surface activation markers of human platelets. Diluted whole blood was treated with safflower extract or compounds found in safflower extract for 1 h, stained with fluorescent antibody, and stimulated with ADP. After platelet fixation, the content of activation markers on the platelet membrane was analyzed by flow cytometry. * *p* < 0.05 when compared to the control group. # *p* < 0.05 when compared to the ADP treatment group.

Glycoprotein IIb/IIIa (also called CD41/CD61, PAC-1 epitope or integrin αIIbβ3) on the platelet membrane is mainly produced after stimulation of platelets by ADP and can combine with fibrinogen to cause platelet aggregation. In addition to the above intraplatelet signal transduction tests, we finally wanted to investigate whether safflower extract and its compounds caused changes in glycoprotein expression on the platelet membrane. In order to simulate the living conditions, whole blood was used for drug treatment and staining analysis, and platelets were selected on a flow cytometer for membrane protein analysis. Although the platelet cell population accounts for less than 1% of blood samples, these glycoproteins on the platelet membrane can still be found to be altered by ADP stimulation (Supplementary Figure S1). In whole blood treatment with ADP, CD61 and PAC-1 production on the platelet membrane was significantly increased. However, when whole blood was treated with safflower extract or its compounds, CD61 on the platelet membrane decreased slightly only in the blood treated with safflower yellow A, but no significant changes were found in the other groups (Figure 6). In contrast, safflower extract or all its compounds inhibited the expression of PAC-1 on the ADP-induced platelet membrane (Figure 6). Combined with the above research results, safflower extract or its compounds can inhibit a continuous signal transduction pathway induced by ADP in platelets, thus affecting the formation of related glycoproteins on the membrane and thereby inhibiting platelet aggregation.

## 3. Discussion

**Figure 7:**
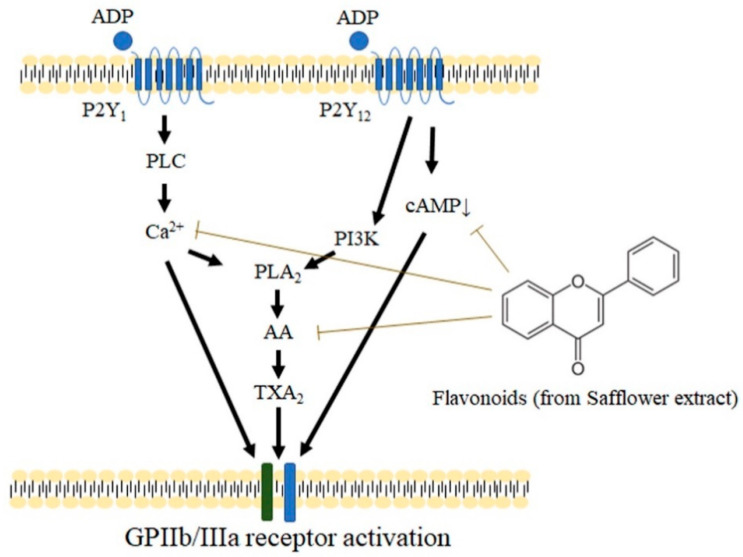
Schematic diagram of the mechanism of safflower extract inhibiting platelet aggregation. The compounds found in safflower extract inhibit the activation of calcium ions in platelets and the transduction of cAMP pathways caused by ADP, thereby inhibiting the expression of TXA_2_ in platelets, the activation of membrane protein GPIIb/IIIa, and the aggregation of platelets.

Our results indicated that safflower extract could inhibit platelet aggregation mainly in the following aspects: ADP receptor transduction and expression of PAC-1 glycoprotein on the platelet membrane, calcium ion activation, and the cAMP, AA, and TXA2 contents regulated by ADP in intracellular platelets were all significantly inhibited by safflower extract (Figure 7). These results suggest that safflower extract extensively inhibits platelet aggregation, though not specifically, in the process of platelet activation. Its curative effect as a traditional Chinese medicine for promoting blood circulation and removing blood stasis may also be related to its inhibition of platelet activation, thereby inhibiting the formation of thrombi.

Although safflower has the effect of promoting blood circulation and removing blood stasis, in vivo experiments were mostly conducted with various compounds of safflower extract as materials. Oral feeding carthamins yellow at 100 mg/Kg and 200 mg/kg in living rats can significantly reduce blood fluidity [30]. In rats, sublingual intravenous injection of 1.5, 3.0, and 6.0 mg/kg of hydroxysafflor yellow A significantly reduced thrombosis, while the treatment of 3.0 and 6.0 mg/kg of hydroxysafflor yellow A significantly reduced the infarct size [31]. In addition, 200 mg/kg/d safflower yellow or hydroxysafflor yellow A in diet-induced obese mice can effectively inhibit fat mass by 57.8–61.6% [32]. Although it has been documented that safflower extract can be converted into substances in liver metabolism [3,4], the concentrations of either safflower extract or these compounds found in safflower extract in the blood and their half-life in the blood after administration are still unknown. In any case, the concentration of the compounds used in the in vitro experiment was similar to the dose used in this study [31].

The compounds found in safflower extract used in this study, whether hydroxysafflor yellow A, safflower yellow A, or luteolin, are all converted into flavonoids during metabolism. Flavonoids can be found in many green vegetables or fruits, and their effects have been extensively studied. Because of their unique benzene ring structure, flavonoids are believed to have a strong antioxidant effect. Studies have shown that they inhibit the metabolic pathway of AA (including PLA2 and COX2) and modulate the activation of transcription factors involved in inflammation, such as NF-κB, GATA-3, and STAT-6 [33,34]. In terms of anticancer research, flavonoids have been found to regulate several important factors related to the growth of cancer cells, such as epidermal growth factor receptors, platelet-derived growth factor receptors, vascular endothelial growth factor receptors, and cyclin-dependent kinases [35,36]. However, flavonoids are not well absorbed by the human body, so even though they have many effects in in vitro studies, except for some evidence of anticancer effects [37,38], none of them have significant clinical effects in the treatment of anti-inflammatory or cardiovascular diseases [39].

In addition to their anti-inflammatory and anticancer effects, flavonoids have also been shown to directly bind to TXA2 receptors to directly inhibit platelet activation [40]. Based on the above research results, when the AA metabolic pathway is inhibited by flavonoids, the production of precursors is inhibited and thus indirectly affects the production of TXA2 in platelets. Concordant with this study, almost all of the various signaling transmitters regulated by ADP, whether calcium ions or cAMP, were affected by safflower extract, suggesting that safflower extract has a wide range of targets and thus has anti-inflammatory and anticancer effects and efficacy against cardiovascular diseases. However, the various compounds of safflower extract are mainly metabolized in liver tissue to produce flavonoids. Therefore, the importance of other metabolites or degradants produced by each compound of safflower extract in inhibiting platelet agglutination cannot be excluded, in addition to the unique structure of flavonoids in the various compounds of safflower extract [41].

In summary, we found that safflower extract and several safflower extract compounds can inhibit ADP-regulated human platelet aggregation. The production of calcium ions, cAMP, AA, and TXA2 regulated by ADP receptors, whether P2Y1 or P2Y12, would be inhibited by safflower extract or compounds found in safflower extract. We further found that the formation of the PAC-1 complex, a glycoprotein involved in the aggregation of platelet membranes, was also inhibited by safflower extract or several safflower extract compounds. Therefore, the use of safflower extract in traditional Chinese medicine as a way to promote blood circulation and remove blood stasis may be related to its inhibition of platelet aggregation.

## 4. Materials and Methods

### 4.1. Preparation of the Aqueous Extract of Safflower

Safflower powder was purchased from Shun Ten Pharmaceutical Co., Ltd. (New Taipei City, Taiwan). A total of 10 g of safflower powder was dissolved in 100 mL of distilled water, boiled, and filtered. The water-soluble safflower extract was sterilized by an autoclave and then stored at 4 °C.

### 4.2. Preparation of Platelet-Rich Plasma (PRP), Platelet-Poor Plasma (PPP), and Purified Platelets

Human blood was obtained from healthy adults through venipuncture in a vacuum tube containing sodium citrate. All participants who participated in this study provided informed consent, and this study was approved by the Taipei Tzu Chi Hospital Institutional Review Board. The obtained blood was centrifuged at 180× *g* at room temperature for 20 min to obtain PRP. The PRP was centrifuged at 1500× *g* for 15 min, and the PPP and isolated platelets were obtained in the supernatant and sediment, respectively. The isolated platelets were resuspended in modified calcium-free Tyrode buffer for subsequent tests.

### 4.3. Measurement of Platelet Aggregation

The platelet processing of safflower extract and the measurement of platelet aggregation were performed using the light transmittance aggregometer platform (PAP-8E, Platelet Aggregation Profiler, Bio/Data Corporation, Horsham, PA, USA) with adjustable temperature and continuous stirring. PRP was treated with safflower extract, hydroxysafflor yellow A (ChemFaces Biochemical Co., Ltd. Hubei, China), safflower yellow A (ChemFaces), or luteolin (ChemFaces) at 37 °C for 1 h. After setting the reference value of each sample with PPP, PRP was treated with 100 μM A2P5P (Santa Cruz Biotechnology, Santa Cruz, CA, USA) or clopidogrel (Santa Cruz Biotechnology) for 3 min. Finally, 10 μM ADP (Sigma-Aldrich, St. Louis, MO, USA) was added, and the aggregation reaction was monitored for 6 min.

### 4.4. Detection of Calcium Ions and cAMP in Platelets

PRP was treated with safflower extract or compounds found in safflower extract at 37 °C for 1 h, after which 3 μM Fura-2AM (Molecular Probes, Invitrogen, Carlsbad, CA, USA) was added for 45 min, and the isolated platelets were obtained by centrifugation. The method of measuring calcium ions has been described previously [42]. For the measurement of cAMP, isolated platelets were obtained from control or safflower extract-treated PRP by centrifugation, and then 10 μM forskolin and 10 μM ADP were added and incubated at 37 °C for 5 min. After washing with PBS, the cells were destroyed with 0.1 N HCl, and the cell extracts were obtained by centrifugation. cAMP analysis was performed using EIA technology and in accordance with the manufacturer’s instructions (cyclic AMP EIA Kit, Cayman Chemical, Ann Arbor, MI).

### 4.5. Determination of Thromboxane B2 (TXB2) and Arachidonic acid (AA) in Platelets

PRP was treated with safflower extract or compounds found in safflower extract for 1 h, and isolated platelets were obtained by centrifugation. Then, 3 μM fibrinogen and 10 μM ADP were added and placed at 37 °C for 3 min for stimulation. The sample was then placed in dry-ice-cooled ethanol to quickly freeze the sample to stop the reaction. The content of TXB2 in each sample was measured in accordance with the manufacturer’s instructions (Cayman Chemical). Alternatively, isolated platelets obtained by the treatment of safflower extract or compounds found in safflower extract with 10 μM ADP and fibrinogen stimulation for 30 min were centrifuged, and the supernatant was obtained to measure the content of AA (Cusabio Biotech, Hubei, China).

### 4.6. Measurement of Platelet Surface Activation Markers

The obtained blood was diluted with PBS at a ratio of 1:2. The diluted blood was treated with safflower extract or compounds found in safflower extract for 1 h and then treated with fluorescent antibodies (PE-conjugated CD61 and FITC-conjugated PAC-1, BD Pharmingen, San Jose, CA, USA) with 20 μM ADP at room temperature for 20 min. The diluted blood samples were immediately fixed with 400 μL of 1% formaldehyde/PBS, and the expression of surface antigen was analyzed by flow cytometry (FACScan, Becton Dickinson, Flanklin Lakes, NJ, USA).

### 4.7. Statistical Analysis

Each experimental data point was obtained from at least three samples. The experiment was performed with at least three replicates and presented as ±SD of the mean. Statistical analysis was performed using one-way ANOVA and Dunnett’s posttest. A *p*-value of less than 0.5 between groups was considered significant.

## Data Availability

All data used to support the results of this study are included in the article.

## Supplementary Materials

The following are available online at https://www.mdpi.com/article/10.3390/plants10061192/s1, Figure S1: Effects of safflower extract on the expression of CD61 and PAC-1 of human platelets.

## Abbreviations

TXA2	thromboxane A2
cAMP	cyclic AMP
PRP	platelet-rich plasma
PPP	platelet-poor plasma
TXB2	thromboxane B2
AA	arachidonic acid
