# Supplementary figures and images for: Safflower Extract Inhibits ADP-Induced Human Platelet Aggregation
Source: Plants (Basel). 2021 Jun 11;10(6):1192. doi: 10.3390/plants10061192 (PMC8230796; doi:10.3390/plants10061192)

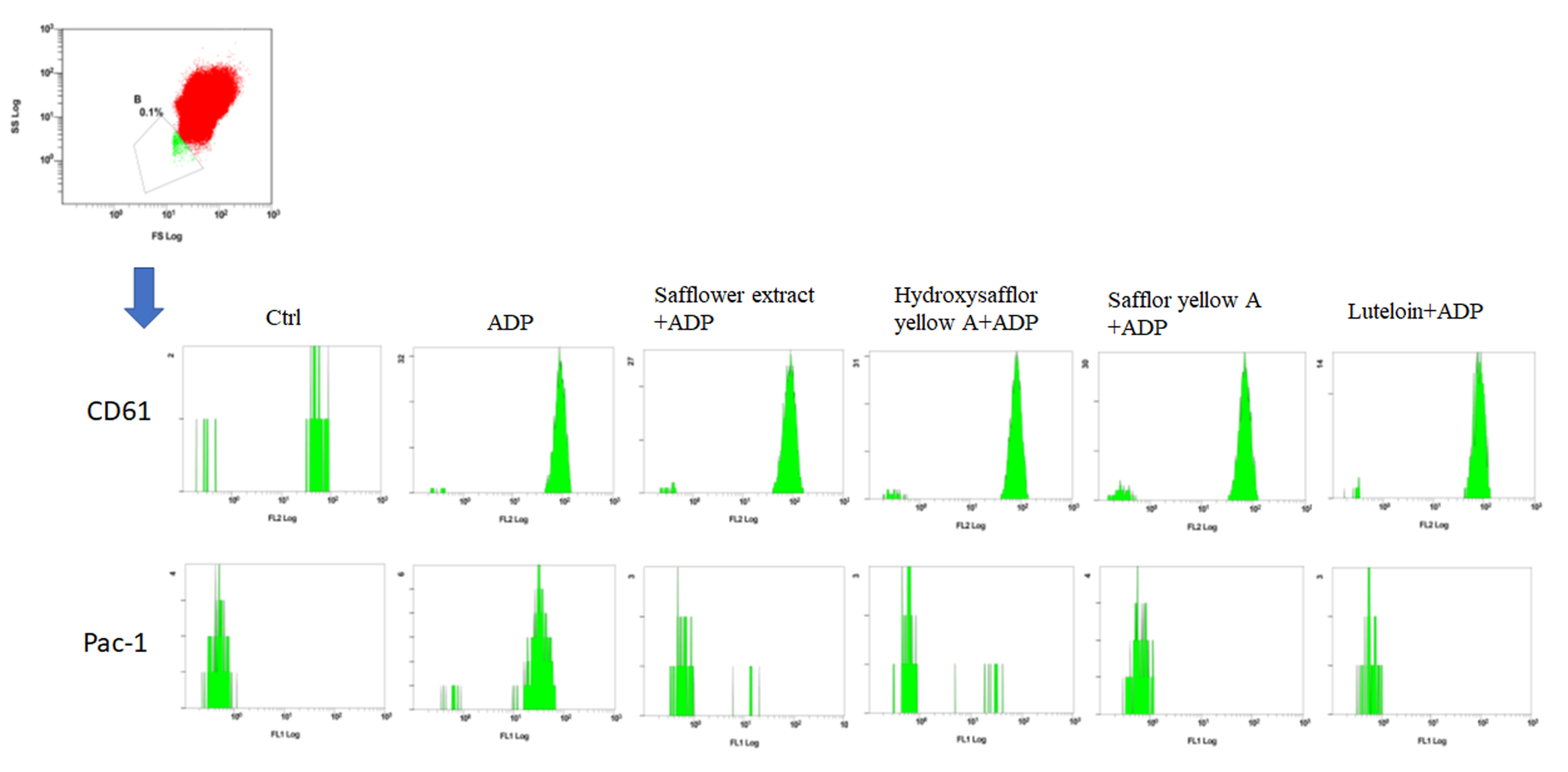

Supplement: Supplementary file 1 [file plants-10-01192-s001.zip › plants-1237816-supplementary.jpg]
